# Thermogravimetry and Mass Spectrometry of Extractable Organics from Manufactured Nanomaterials for Identification of Potential Coating Components

**DOI:** 10.3390/ma12223657

**Published:** 2019-11-06

**Authors:** Per Axel Clausen, Vivi Kofoed-Sørensen, Asger W. Nørgaard, Nicklas Mønster Sahlgren, Keld Alstrup Jensen

**Affiliations:** 1 National Research Centre for the Working Environment, DK-2100 Copenhagen, Denmark; vks@nrcwe.dk (V.K.-S.); awnq@novonordisk.com (A.W.N.); nms@nrcwe.dk (N.M.S.); 2 Novo Nordisk, DK-2760 Måløv, Denmark

**Keywords:** engineered nanomaterials, surface coating, TGA, GC-MS, LC-MS, MALDI

## Abstract

Manufactured nanomaterials (MNMs) often have a surface-chemical modification in order to tailor their physicochemical properties, including also powder properties and miscibility. Surface-chemical modifications may influence the toxicological properties of the MNM, but the specific chemistry and extent are rarely described in detail in suppliers’ technical data sheets. Chemical and quantitative information on any surface-chemical treatment, coating and functionalization are required for chemicals registration in Europe. Currently there is no globally accepted and documented approach to generate such data. Consequently, there is a continued research need to establish a structured approach to identify and quantify surface-chemical modifications. Here we present a tiered approach starting with screening for mass-loss during heating in a furnace or thermogravimetric analysis (TGA) followed by solvent extraction, and analysis by several mass spectrometry (MS) techniques depending on the target analytes. Thermal treatment was assumed to be able to quantify the amount of organic coating and MS was used to identify the extractable organic coatings after pressurized liquid extraction (PLE) using methanol at 200 °C. Volatile organic compounds in extracts were identified with gas chromatography and MS (GC-MS), non-volatile organic compounds with liquid chromatography MS (LC-MS), and polymeric compounds with matrix-assisted laser desorption ionization time-of-flight MS (MALDI-TOF-MS). The approach was demonstrated by analysis of 24 MNM, comprising titanium dioxide, synthetic amorphous silica, graphite, zinc oxide, silver, calcium carbonate, iron oxide, nickel-zinc-iron oxide, and organoclay. In extracts of 14 MNMs a range of organic compounds were identified and the main groups were silanes/siloxanes, fatty acids, fatty acid esters, quaternary ammonium compounds and polymeric compounds. In the remaining 10 MNMs no organic compounds were detected by MS, despite the fact an organic coating was indicated by TGA.

## 1. Introduction

Manufactured Nanomaterials (MNMs) may be chemically modified by surface coating or functionalization depending on their technical applications, see e.g., Baraton [1], Gref et al. [2], Kinge et al. [3], Basiruddin et al. [4], and Sperling and Parak [5]. Surface coatings may change the physical, chemical and toxicological properties of the MNM as compared to that of the core material and prevent them from agglomeration [6] before incorporation into intermediates and final products such as polymeric matrices [7], biomedical products [5,8], or electronics [9]. Surface chemical modification, however, is not unique to MNMs. It has been used for a long time for dispersion and stabilization of, for example, pigments in cosmetics and paints [10]. Therefore, the approach presented herein is also of general relevance and applicability.

Surface-chemical coatings of MNM may occur as either solid or molecular substances of inorganic or organic nature or a combination of both, as described in detail by Atluri and Jensen using silica as an example [11]. The strategy for coating or surface functionalization of core/shell MNMs [12], generally falls into four classes [5]: (1) ligand-like binding by chemisorption, (2) electrostatic adsorption, (3) covalent binding, (4) non-covalent, affinity-based receptor-ligand systems (adsorption). Consequently, removal of unknown coating molecules with unknown binding type to the MNM surface for further chemical analysis requires harsh removal (extraction) conditions which in turn may also degrade the coating molecules.

A number of scientific papers identify surface chemistry as important piece of information in order to assess the environmental and toxicological effects of MNMs [13,14,15]. Surface chemistry is also important in established risk categorization and control banding tools [16] and recently in proposed scientific systems for hazard grouping [17,18]. More importantly, reporting of any surface-chemical modifications is currently requested for chemicals registration of substances nanoforms and proposed as an element for read-across and Quantitative Structure Activity Relationships in REACH [19,20]. However, even though surface-chemical modifications may be reported more accurately in the future due to regulatory requirements, it is anticipated that there will be an emerging need for a harmonized structured approach for identification and quantification of surface chemical modifications, both from manufacturers and downstream users and authorities. The hitherto flexibility in the regulatory request to report surface chemical modifications for only chemically bound well-defined substances may be one of the key reasons that generally none or imprecise information on chemical surface modifications is given in manufacturers technical and material safety data sheets. This has been documented for chemicals in cleaning products [21], in addition to a lack in toxicity and physicochemical property information [22]. This leads to an apparent critical uncertainty in material identification and downstream risk assessment, as well as in conclusions drawn from toxicological testing in scientific papers. Based on the above discussion, there is an urgent need to establish operational standard methods and technical guidance on how to identify surface chemical modifications of MNMs and how to quantify the amount. 

The surface chemistry of MNMs may be characterized by a number of methods, including i.e., nuclear magnetic resonance (NMR), Fourier transform infrared spectroscopy (FTIR), mass spectrometry (MS), X-ray photoelectron spectroscopy (XPS), combustion elemental analysis, and Auger electron spectroscopy [23,24,25,26,27]. However, application of these methods may be challenging and surface coatings cannot be identified and quantified by many of these methods [27]. A recent book chapter also reviewed surface chemical analysis methods applied to MNM and gives some educational examples using X-ray photoelectron spectroscopy (XPS) and time-of-flight secondary ion mass spectrometry (TOF-SIMS) [28]. In spite of the obvious advantages of using MS and hyphenated MS techniques for the analysis of MNM surface coating only few original papers are published using these methods, even-though Zhang and Yan [24] concluded that analysis using liquid chromatography coupled with MS (LC-MS) after ligand cleavage is the only method for qualitative and quantitative analysis of multiple functionalized MNMs. However, for one particular type of MNM, namely monolayer coated gold nanoparticles, MS has attracted much attention for identification and quantification of individual coating compounds [29]. For other types of coated MNMs only a few sporadic studies using MS for identification and quantification of coating compounds are published (see Table 1).

It is a specific analytical challenge that the composition and structure of surfaces are difficult to control [42] which impacts the reproducibility of both the identification and quantification of surface coatings of MNMs. It was proposed to use provenance information as a tool for addressing MNM reproducibility challenges [42] and this information is also important for identification of a specific surface coating. The batch-to-batch inhomogeneity of MNMs was shown to be reflected in different physicochemical properties including impurities and formation of reactive oxygen species (ROS) [43].

Here we present an approach to identify and quantify extractable organic coatings of MNMs using different MS techniques and mass-loss during thermal treatment using either loss-on-ignition (LOI) corrected for water-content (WC) or TGA to quantify the amount of coating. The chemical analytical approach was based on extraction using pressurized liquid extraction, and two separation techniques combined with MS, GC-MS and LC-MS, which were used for analysis of volatile and non-volatile organic compounds, respectively. Finally, MALDI-TOF-MS was used for analysis of non-volatile and polymeric organic compounds.

## 2. Materials and Methods

### 2.1. Manufactured Nanomaterials

Table 2 summarizes the manufactured nanomaterials (MNMs) that were analysed in this study. The samples were obtained and analysed as part of our participation of the EU-projects FP7 NANODEVICE, EAHC NANOGENOTOX, and FP7 NANoREG as well as the Danish NANOPLAST, and the Danish Nanosafety Centre. The final characterization and protocol development [44] was completed as part of the FP7 NANoREG project. All MNM were analysed with thermogravimetric analysis (TGA) and when it indicated >1% mass-loss due to apparent organic coating, i.e., corrected for initial water loss, the MNM was selected for extraction and MS analysis (Table 2; for more details see Table S1 in the Supplementary Materials). 

### 2.2. Chemicals

Methanol (99.9% Chromasolv) was obtained from Fluka (Sigma-Aldrich Denmark, Copenhagen, Denmark), acetonitrile (LC-MS Chromasolv) was from Riedel-de-Haën (Sigma-Aldrich Denmark, Copenhagen, Denmark) and water was Milli-Q-filtered water (Elga, Krüger Aquacare, Glostrup, Denmark). Ammonium acetate (99.999 trace metal basis), 2,5-dihydroxybenzoic acid (>99 %, matrix substance for MALDI-MS), dextran (MW ~ 6000), and polyethylene glycol 1500 were obtained from Sigma-Aldrich (Saint Louis, MO, USA).The following authentic standards were used: tetramethoxysilane, trimethoxymethylsilane, trimethoxyoctylsilane, hexamethylcyclotrisiloxane, octamethylcyclotetra- siloxane, 2-pyrrolidone, tetradecanoic acid methyl ester, pentadecanoic acid methyl ester, hexadecanoic acid methyl ester, octadecanoic acid methyl ester, tetradecanoic acid, hexadecanoic acid, and octadecanoic acid (all >99% purity; Sigma-Aldrich, Saint Louis, MO, USA). Alkyltrimethylammonium chloride with C_16_ (>98%) and C_18_ (>95%) alkyl chains, dimethyldialkylammonium chlorides or bromides with C_14_ (>97%), C_16_ (97%) and C_18_ (>97%) alkyl chains, and benzalkonium chloride with a C_12_ alkyl chain were also obtained from Sigma Aldrich.

### 2.3. Thermogravimetric Analysis

The thermogravimetric analysis (TGA) of the MNMs was performed to quantify the adsorbed water content and a possible organic coating using a STA 449 F3 Jupiter instrument (Netzsch-Gerätebau, Selb, Germany). The TGA was performed according the NANoREG standard operating procedure for TGA [44]. The TGA was carried out in an oxygen atmosphere (air) to insure complete oxidation of the organic compounds. The temperature program for all samples was heating from 25 °C to 50 °C at 10 °C/min and hold for 1 min, then heating to 100 °C at 2.5 °C/min and hold for 10 min, then heating to 800 °C at 2.5 °C/min and hold for 1 min. The sample holders (crucibles) used for the TGA measurements were made of alumina (Al_2_O_3_) and had a volume of 3.4 mL. Sample masses were 10–60 mg and no samples were conditioned to equilibrate with known air humidity. Data was corrected for buoyancy. TGA was performed in triplicates.

### 2.4. Water Loss and Loss on Ignition (LOI) by the Laboratory Furnace Method

Water loss was determined by weighing and heating at 110°C overnight in a T5028 oven (Heraeus Holding GmBH, Hanau, Germany). The crucibles were cooled in an exsiccator (20 min) immediately after oven treatment and then weighed. After weighing the crucibles were placed in a high temperature oven (Nabertherm N31/H, Nabertherm GmbH, Lilienthal, Germany) at room temperature and heated over 12 h to 1050 °C and hold for one hour (6 h and 500 °C for the CaCO_3_ MNM due to their decomposition at higher temperature). At 175 °C the crucibles were transferred to exsiccator, cooled (20 min) and finally weighed. Sample masses were approximately 5 g. Measurements were performed in triplicate.

### 2.5. Pressurized Liquid Extraction

Pressurized liquid extraction (PLE) was performed using an ASE 200 system (Dionex, Thermo Fisher Scientific, Hvidovre, Denmark). The samples (300–500 mg) were weighed into 5 mL extraction cells which were filled with Ottawa sand (Fisher, Hampton, NH, USA) to reduce the dead-volume. The samples were extracted with methanol at 200 °C and 140 bar (heating for 9 min, static extraction for 10 min, and flushing by 25% of the volume). The extract (2 mL) was centrifuged at 20,000 G and 22 °C for 30 min and the supernatant was used for the succeeding analysis. If the sample contained very small particles which were not removed by the centrifugation it was necessary to filter the sample before analysis. PLE was performed in 2–4 replicates and blank extracts were included in each series to make sure that no compounds found in the extracts of the MNM were a part of the background.

### 2.6. Gas Chromatography-Mass Spectrometry

Gas chromatography-mass spectrometry (GC-MS) analysis was performed using a SCION TQ MS instrument (termed MS throughout the article, Bruker Daltonics, Billerica, MA, USA). The PLE extract was injected directly (1 µL) into the GC-MS which was equipped with a 30 m VF-5ms capillary column with a diameter of 0.25 mm and 0.25 µm stationary phase containing 5% phenyl poly dimethylsiloxane (Agilent Technologies, Santa Clara, CA, USA). The column flow was 1 mL/min helium and the injector temperature was 250 °C. The GC oven program started by keeping the temperature at 40 °C for 4 min and then increasing it by 4 °C/min to 120 C and 8 °C/min to 250 °C and held for 10 min. The transfer-line and source temperature was 275 °C. The mass spectrometer was operated in electron ionization mode (EI, 70 eV) using full-scan mode (m/z 50–500). Identification of the organic compounds was performed by MS Data Review, Version 8.0.1 (Bruker), AMDIS version 2.70 May 13, 2011 and NIST/EPA/NIH Mass Spectral Library Version 2.0 g, May 19, 2011 (NIST, Gaithersburg, MD, USA). The following GC-MS properties of authentic standards were used for identification: Retention time (t_R_), peak shape, and mass spectrum. Semi-quantification was performed by MS Data Review with external calibration. Four major fatty acid esters and three fatty acids were identified by authentic standards and the remaining by the mass spectra and retention times. All fatty acids and fatty acid methyl esters were semi-quantified in equivalents of octadecanoic acid and methyl octadecanoate, respectively. The un-identified silanes, denoted with a “?” in Table 3, had consistently silanes/siloxanes as compounds with the highest score in the NIST database. Tetramethoxysilane was semi-quantified by its authentic standard and all unidentified silanes were semi-quantified in equivalents of octamethylcyclotetrasiloxane. All other compounds were semi-quantified in equivalents of octadecanoic acid methyl ester. The semi-quantitative result is the average of the results of each of the performed extractions. 

### 2.7. Liquid Chromatography-Mass Spectrometry

Analysis with liquid chromatography combined with mass spectrometry (LC-MS) were performed using an Agilent 1200 high performance liquid chromatograph coupled to a Bruker microTOF-Q quadrupole time-of-flight mass spectrometer (termed QTOF throughout the article). All LC-MS analyses except for quaternary ammonium compound (QAC) coatings of the organoclays were performed using the following condition: 5 µL of PLE extract was injected directly onto a ZORBAX Eclipse Plus C18 (1.8 µm, 2.1 × 50 mm) column (Agilent) held at 30 °C and a flow of 0.3 mL/min. The mobile phase was water as solvent A and acetonitrile with 1 mM ammonium acetate as solvent B. The elution program consisted of a linear gradient from 90% to 20% of solvent A within 12 min and held for 4 min. PLE extracts of the organoclays containing QAC were injected directly (1 µl) into the LC-MS system and separation was performed with a ZORBAX Eclipse XDB-C18 (1.8 µm, 4.6 × 50 mm) column (Agilent) at constant 80 °C and a flow rate of 0.2 mL/min. The mobile phase was 2-propanol with 0.1% formic acid as solvent A and pure water as solvent B. The elution program consisted of a linear gradient from 60% to 90% of solvent A within 22 min. Ionization of analytes was performed using electrospray ionization (ESI) in the positive mode. Temperature of source and nitrogen dry gas was 190 °C. The capillary voltage was −4.5 kV and the measurement range was m/z 100–2500. The polyethoxylates of NM-103 was semiquantified compared to polyethylene glycol 1500. The identification of the QAC was based on the exact mass of the molecular ions (within 3 ppm) and the retention time. External calibration curves using the molecular ions as quantifiers were made from known standards of six different QACs. 

### 2.8. Matrix-Assisted Laser Desorption Ionization Time-of-Flight Mass Spectrometry

Matrix-assisted laser desorption ionization time-of-flight mass spectrometry (MALDI-TOF-MS) was performed using an Autoflex II (Bruker Daltonics). Sample preparation was performed by the dried droplet method. The MNM dispersed in water was mixed (50:50) with the matrix solution (saturated 2,5-dihydroxybenzoic acid in water/acetonitrile; 80:20 v/v). 1 µL of the sample/matrix mixture was deposited on the MALDI steel target and dried at room temperature. Positive ion MALDI-TOF-MS spectra (256 summed acquisitions) were acquired using both delayed-extraction (90 ns) and reflector (21 kV) modes, with accelerating voltage at 19 kV, nitrogen laser (337 nm) with 30% power, and the low mass range mode set at m/z 50–5000. Dextran with MW ~6000 was used for mass calibration of the MALDI-TOF-MS. The identification of the polymeric compounds from the MALDI-TOF-MS results was based on pattern recognition and literature (see Extraction, separation, and mass spectrometric analysis of NRCWE-009 and NM-300K in the Results and Discussion section).

## 3. Results and Discussion

### 3.1. Thermogravimetric Analysis (TGA)

**Figure 1:**
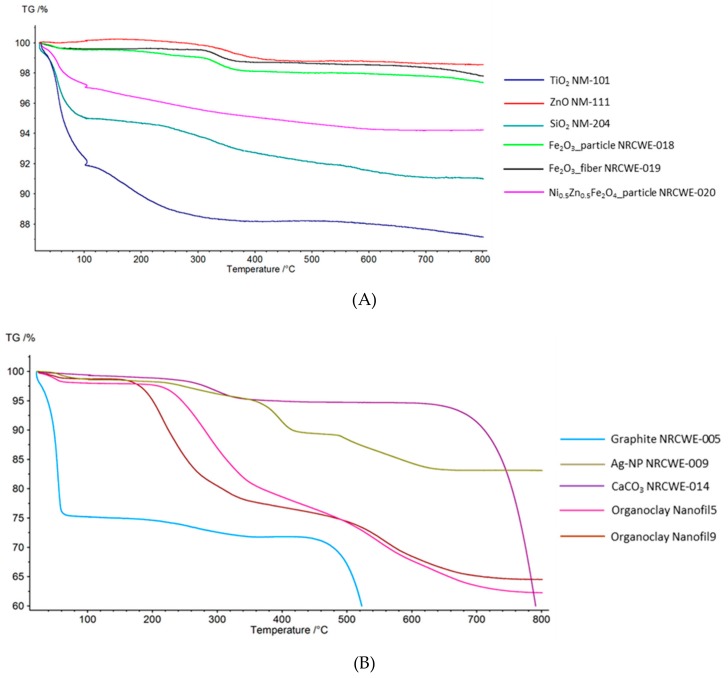
TGA curves of the wt.% mass loss of different types of MNM potentially having an organic coating. (**A**) TGA curves that fit into a relative mass range of 86–100 wt.%, (**B**) TGA curves for MNM with large mass loss. The small negative peak at 100 °C is due to the mass loss during the 10-min period of the TGA program with constant 100 °C.

TGA curves for the selected MNMs (Table 2) are shown in Figure 1. Water loss was defined as mass loss in the interval 25–110 °C (including the 10 min constant 100 °C period of the TGA program) and loss of associated organic material or coating as mass loss from 110 °C to 800 °C except for the graphite that decomposed at 450–600 °C, the carbonates that decomposed at 700–800 °C, and in cases where a weight gain is observed at the end of the TGA curve.

The titanium dioxide (TiO_2_) MNM (UV-Titan M111, NM-101, NM-103, NM-104) showed similar patterns with an almost continuous and significant mass loss from 25–800 °C which might be a combination of loss of water and associated organic material or coating. The total mass-losses observed for the TiO_2_ materials in this study were generally higher than the values reported by the suppliers (Table 2). NM-103 was, according to manufacturer’s information, expected to have 2 wt.% coating of dimethicone, which is a mixture of fully methylated linear siloxane polymers [–(CH_3_)_2_SiO–]_x_ end-blocked with trimethylsiloxy units [–(CH_3_)_3_SiO–] [54]. NM-104 was, according to published results [47], expected to have 1 wt.% coating of glycerin. The supplier did not report any organic surface chemical treatment or coating on M111 and NM-101. The manufacturer reports M111 to consist of >85 wt.% TiO_2_ and that it is coated with alumina. NM-101 was expected to have the largest amount of coating based on our previous TGA analysis identifying ca 8 wt.% coating [46], but it is reported to be a pure TiO_2_ nanomaterial. Elemental analysis of NM-101 showed the presence of 0.09 wt.% Al, 0.29 wt.% Si, 0.27 wt.% P, 0.22 wt.% S, and >0.1 wt.% Na [46,55]. The Si-concentrations are between that in NM-103 (0.68 wt.%) and NM-104 (0.18 wt.%). The chemical composition of NM-101 can indicate potential presence of sulfate and/or phosphates and/or silane/siloxanes. Moreover, the material consists of very small crystallites of ca. 7 nm size occurring mainly in dense aggregates [46,55]. This could result in a high nanoporosity where water and other substances from synthesis and post-treatments could be trapped and released slower during thermal treatment.

The synthetic amorphous silica (SiO_2_) MNM (NM-204, NRCWE-008) both had significant mass losses below 110 °C suggesting that these samples contained adsorbed water. However, NM-204 had a two-step mass loss whereas NRCWE-008 had a more continuous mass loss up to 650 °C. This suggests presence of an organic compound in NM-204, which may be a functional coating. However, the elemental composition of NM-204 has also shown the presence of ca. 0.2 wt.% Na and 0.2 wt.% S and 0.5 wt.% Al, that may occur in thermally degradable Na_2_SO_4_.nH_2_O and AlO(OH), which were identified in other comparable silica MNM [48]. Previous analyses has suggested <1 wt.% coating for NM-204 [55], which is much lower than the 3 wt.% indicated by the mass-loss between 110 and 700°C (Table 2).

The graphite MNM (NRCWE-005) appeared first to possibly release water in large amount (25 wt.%) and then to release possible organic material (2.7 wt.%) in one step from 110-450 °C. However, such a dramatic mass loss at 50°C may suggest that this mass-loss was not due to water release. Finally the largest mass-loss was observed at 450–600 °C, which is ascribed to decomposition of the graphite (burning), leaving a residual mass of <20 wt.%. Bulk chemical analysis showed that NRCWE-005 contains 27 wt.% Fe and 1 wt.% Ca and residues after combustion by TGA in air was reported to contain iron oxide and quartz (unpublished results). Consequently, the results show that the NRCWE-005 sample tested contained less graphite than reported by the supplier (93 wt.% graphite).

The zinc oxide (ZnO) MNM (NM-111), which is known to be coated using the silane coupling agent triethoxyoctylsilane, had no observed mass loss at 25–110 °C and thus contained no measureable amounts of adsorbed water. Actually, a mass gain of 0.34 wt.% was observed at 25–170 °C and then a two-step decrease. The mass gain was not due to buoyancy but could not at first be explained. Surface-chemical analysis by X-ray Photoelectron Spectroscopy showed that the surface is strongly dominated by oxygen with an O/Zn ratio of ca. 5.07 and has a Si/Zn ratio of 0.75 (n = 7) [51]. Consequently, the ZnO mass-gain is unlikely be due to uptake of oxygen to balance a potentially non-stoichiometric composition of the ZnO. The weight gain is rather related to a chemical reaction (oxidation with no degradation) in the surface chemical treatment. When the oxidation process starts degrading the coating the mass starts to decrease. Due to the mass gain the coating amount was measured as the mass-loss from 25–800 °C and constituted about 2 wt.%. This is somewhat more than the previously reported mass-loss of coating of 1% by TGA [51]. 

The Ag MNM, NRCWE-009, was associated with 15 wt.% evaporable and combustible organic material that decomposed in several steps (Figure 1B), which indicates the presence of one or more organic compounds with complex molecular chemistry. NRCWE-009 was originally donated by the manufacturer to NRCWE as a Ag nanoparticle powder with limited information as a special research request. The Ag MNM, NM-300K, is an aqueous colloidal dispersion with a nominal silver content of 10 wt.%, stabilizing agents consisting of 4 wt.% polyoxyethylene glycerol trioleate (Tagat^®^ TO) [56] and 4 wt.% polyoxyethylene sorbitan mono-laurate (Tween^®^ 20) [53]. The TGA curve showed weight loss in several steps (Supplementary Materials, Figure S1). The water loss was 68 wt.% and the amount of associated organic material was 16 wt.% resulting in residual mass of 17 wt.% of Ag. Thus the water content appeared to be lower whereas the organic material and the Ag content were higher than expected values. This may be due to inhomogeneity in the sample material or water loss during storage in the vial. The ratio of organic mass to Ag mass was in accordance with the previously reported data [53].

The calcium carbonate (CaCO_3_) MNMs (NRCWE-012 to NRCWE-017) all have a small possible water content below 1 wt.%. This is in good agreement with the supplier’s information. For all carbonates the apparent coatings decomposed in two steps. The curves show slow mass losses at 110–250 °C and then a fast mass loss at 250–350 °C (Figure 1B), except for NRCWE-015, where the second step at 250–450 °C is slower than for the other carbonates. The full decomposition of the CaCO_3_ samples into CaO and CO_2_ starts around 650 °C. Use of a TGA program with a final temperature of 1000 °C and complete decomposition showed that the residual mass was not very reproducible for the individual CaCO_3_ samples and varied between 53 wt.% and 58 wt.%, indicating that they were inhomogeneous materials.

The iron oxide (α-Fe_2_O_3_) MNMs (NRCWE-018 and NRCWE-019) both have stepwise but different mass losses, which most likely correspond to the decomposition of an organic coating (Figure 1A). From the morphology showing partially irregular shapes and pores, in particular for NRCWE-019 fibres [57], the material appears to be produced by wet-chemical synthesis. Hence, high-temperature mass losses could also be caused by dehydration of trapped synthesis liquids and/or dehydroxylation due to possible presence of minor amounts of iron oxyhydroxide. Presence of coatings or additives were not reported by the supplier.

The nickel-zinc-iron oxide (Ni/ZnFe_2_O_4_) MNMs (NRCWE-020 and NRCWE-022) both showed continuous mass losses (Figure 1A). If the observed mass loss was due to coating it seems an unusual behavior since one would expect the organic coating to decompose in steps. It could thus be speculated to be due to continuous evaporation and not decomposition. Nogueira et al. [58] also observed a weight gain after 600 °C of NiFe_2_O_4_ and explained it by formation of oxides, however, the iron is already in oxidation state 3 and cannot be further oxidized. Singh et al. [59] also observed a weight gain of Ni_0.5_Zn_0.5_Fe_2_O_4_, however after about 675 °C, but they provided no explanation. Transmission electron microscopy images of these samples show dense agglomerates or aggregates of small nanocrystallites with an average X-ray crystallite size of 10–12 nm [57]. This is a similar appearance as NM-101. It is possible that there is residual water present from synthesis and post synthesis processes in the nanoporous structures of the agglomerates/aggregates. As for NRCWE-018 and NRCWE-019, another possibility is presence of a small fraction of X-ray amorphous oxyhydroxides, which dehydrate and dehydroxylate upon thermal treatment. The presence of coatings or additives was not reported by the supplier.

The organoclay MNMs were known a priori to be coated with large amounts of dialkyldimethyl ammonium compounds (Nanofil 5^®^, Nanofil 8^®^, Nanofil SE3000^®^) or benzalkonium compounds (Nanofil 9^®^) (Table 2). The TGA curves of all organoclays were very similar, with a tendency for Nanofil 9^®^ to start out mass loss before the other organoclays of which the initial mass loss was quite sudden and took place at the same temperature (Figure 1B). This probably reflects the different types of quaternary ammonium compounds (QAC) coatings. The mass loss appeared to be 2-step for all organoclays and may be speculated to be due to two mechanisms: evaporation of decomposition products and combustion of the QAC since the ionic QACs are non-volatile.

### 3.2. Comparison of TGA and the Laboratory Furnace Method for Water loss and Loss on Ignition (LOI)

**Figure 2:**
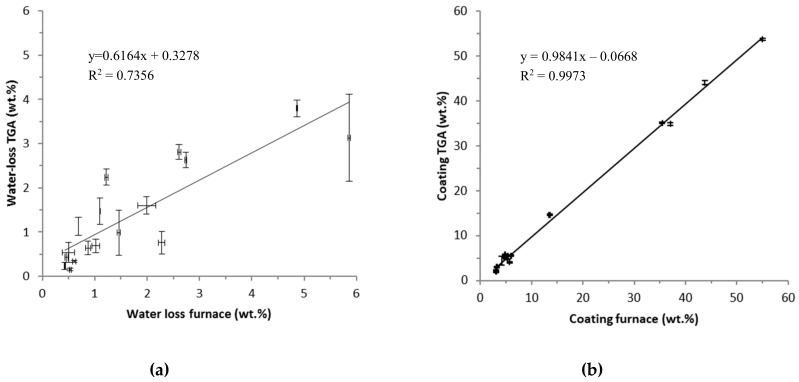
Water loss (**a**) and potential organic coating on MNM (**b**) estimated by TGA versus the furnace method. The error bars represent the standard error of mean, but are not visible for amount of coating.

Figure 2 shows the comparison of water loss and amount of coating as estimated by TGA and the laboratory furnace method, respectively.

The water loss estimated by the applied TGA method gave on average 2/3 of the water loss estimated by the furnace method. However, the data points are somewhat scattered and have large error bars. This indicates that the materials may have varying water contents and/or difficulties in accurate determination of the water contents. Considering the methods, the underestimation of water content by the TGA method may be due to a still too fast heating rate and a too low or too short constant temperature period at 100 °C. The results from the two methods compared much better for the estimated amounts of organic coating as determined from the mass-loss above 110 °C (slope = 1.02, R^2^ = 0.99). However, the good correlation is largely determined by MNM with a higher mass-loss ascribed to coatings while for the MNM with low mass-losses, there is a larger difference between the results obtained by the two methods. This observation may be due to sample inhomogeneity, which is expected to play a greater role in the TGA data due to the much lower mass (10–60 mg) used for TGA analysis as applied in the furnace method (ca. 5 g). Performing TGA with a higher heating rate (10 °C/min from 25–1000 °C) worsened the comparability between the two methods’ estimate of the water content (Slope = 0.32, R^2^ = 0.83, Figure S3).

All together the results show that the heating rate is of particular importance for a proper water content estimation. However, the lower water content estimated by TGA was not reflected in a higher organic mass fraction or total mass loss. The heating rate should potentially be even lower than 2.5 °C/min between 50 and 100 °C or the hold-time at 100 °C should be extended beyond 10 min and/or the dwell temperature should be raised to 110 °C to reach a higher comparability with the furnace method. However, raising the dwell-time and/and dwell-temperature could also result in a risk of partial loss of specific surface chemical coatings, stabilizers, and additives. These issues and considerations are important for analysis of unknown materials.

### 3.3. Extraction, Separation, and Mass Spectrometric Analysis

Pressurized liquid extraction (PLE) was used to extract the organic surface coatings from the MNM. GC or HPLC was used to separate the extracted coating components which were detected and identified using MS (GC-MS) or QTOF (LC-MS). Polymeric compounds in the extracts were detected and identified using MALDI (MALDI-TOF-MS). The applied combinations of techniques for each MNM are shown in Table 3. The last column of Table 3 shows the fraction (wt.%) of TGA measured amount of coating explained by extraction and semi-quantitative GC-MS or LC-MS results. This measure is used to assess the validity of the MS results in order to conclude whether the identified organics are part of a coating or whether it could be contaminants. The larger the explained fraction, the more certain it is that the identified organics are part of a coating. MALDI-TOF-MS is not quantitative and was solely used for identification of polymeric compounds (2^nd^ last column of Table 3). 

**Figure 3:**
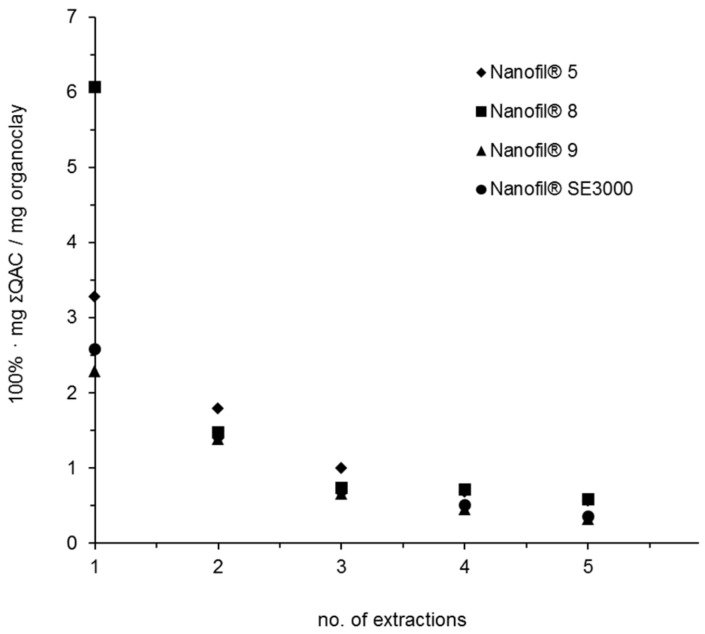
The extraction of total QAC (ΣQAC) of five consecutive extractions of the organoclays. Each point represents the average of three extractions.

The efficiency of extraction of QACs from the organoclays is an illustrative example (Figure 3). In the first extraction (no. 1) the amount of ΣQACs extracted explained 5–14 wt.% of the amount of coating estimated by TGA (last column Table 3). After 5 consecutive extractions still only in total 10-22 wt.% of the coating was extracted. The composition of QAC in each extract remained largely the same. One reason for the low recovery may be the ionic binding of the QAC to the clay platelet surface and the reason that anything at all could be extracted may be due to excess QAC compared to the number of ionic sites on the clay surface.

A range of organic compounds associated with the MNMs was measured, however, the main groups were silanes/siloxanes, fatty acids, fatty acid esters, QAC and polymeric compounds. The groups are shown in Table 3 and are termed ΣSilanes/siloxanes, Σfatty acid methyl esters, ΣFatty acids, ΣDilkyldimethylammonium compounds, and ΣAlkyldimethylbenzylammonium compounds, respectively. The types of compounds were mainly dependent on the manufacturer and the type of MNM.

NM-103 was the only TiO_2_ MNM from which a possible organic coating could be extracted and detected by MS methods. NM-103 was according to manufacturer’s information coated with 2% dimethicone (mixture of fully methylated linear siloxane polymers [54]. However, only trimethoxymethylsilane and hexamethylcyclotrisiloxane were identified (using authentic standards) in the extract (Table 3), in addition to two possible silanes which could not be identified in spite that chemical ionization was applied in the MS analysis.

All of them were in low amounts and should not be contained in dimethicone. In the extract were also found a polyethoxylate (repeat unit *m/z* 44) by LC-MS and MALDI-TOF-MS which probably is a non-ionic surfactant used in the manufacturing process or intentionally added as a coating. It seems strange that nothing but tetramethoxysilane (TMOS) could be detected in the extract of NM-104 since published results show that it is coated with 1 wt.% glycerin. However, glycerin may not be well analyzed with GC-MS or be detected with LC-MS and MALDI-TOF-MS. Elemental analysis performed by energy dispersive spectrometry on sample pellets of NM-103 and NM-104 showed a Si-content of 0.68 wt.% and 0.18 wt.%, respectively [46]. Considering, that the molecular mass-fraction of Si in dimethicone is 37.8746 wt.%, 2 wt.% dimethicone would result in ca. 0.76 wt.% Si in NM-103. This suggests that there is no additional Si in this sample than what originates from dimethicone. However, this is not the case when the mass-loss above 110 °C is considered. No organic coatings were observed in the detailed GC-MS and MALDI-TOF analysis of NM-101. Hence, the mass-loss in NM-101 is ascribed to adsorbed water or non-carbonacous compounds in the nanoporosites of the aggregates and/or degradation of potential present sulfates and phosphates above 110 °C.

No organic compounds were found in the extracts of the synthetic amorphous silica (SiO_2_) MNM (NM-204, NRCWE-008), except for tetramethoxysilane (TMOS). However, TMOS is chemically very reactive [60,61] and cannot be a part of the coating. The fact that the content of TMOS in the extracts of NRCWE-008 can be up to five times the TGA estimated amount of coating indicate that it is formed during the extraction using the 200 °C hot liquid methanol. PLE was performed on NRCWE-008 using acetone and dichloromethane and no TMOS could be detected in these extracts. The retention time of TMOS could vary with about 0.5 min and the GC-MS peak shape and the mass spectrum remained to be most important for identification. NM-204 and NRCWE-008 were also investigated using MALDI-TOF-MS but no polymeric compounds were detected. We have no clear explanation for difference in extracted amount of TMOS from NM-204 and NRCWE-008. However, the manufacturers technical data informs that the NRCWE-008 silica has a higher than nominal silicon-content (SiO_x_ where x = 1.2–1.6), which could mean that the silicon is partially charge-balanced by ions from the synthesis process and/or its surfaces are highly hydroxylated.

Table 3 shows that TMOS was present in the extracts of 12 of the investigated MNM, however, in small amounts, except NRCWE-008. This may be an artifact formed by the hot methanol when the MNM is associated with silica which might be speculated to constitute an inorganic coating or simply an impurity. 

For the graphite MNM (NRCWE-005) MS results could only explain 6 wt.% of the TGA mass loss. The main compounds were fatty acids and their methyl esters in addition to small amounts of presumably a couple of silanes and an aromatic compound. One of them was found to be a trimethoxyalkylsilane probably with same identity (mass spectrum and GC-MS retention time) as the conversion product of the coupling agent from the ZnO MNM (NM-111) which supposedly should be trimethoxyoctylsilane (see results for NM-111). However, such a coupling agent is not expected to bind to graphite surfaces [62] so there is probably no purpose of this compound. No polymeric compounds were detected using MALDI-TOF-MS.

For the zinc oxide (ZnO) MNM (NM-111) GC-MS results explained about 10 wt.% of TGA mass-loss and only one type of compound was identified, namely trimethoxyalkylsilane, which appear to be the result of chemical decoupling during the 200 °C PLE methanol extraction of the original coupling agent triethoxyoctylsilane used for the chemical bonded surface functionalization [51]. 

The PLE extraction of the Ag MNM, NRCWE-009, at 200 °C released solely 2-pyrrolidone which is a known thermal degradation product of polyvinylpyrrolidone (PVP) at about 100 °C [63]. PLE of pure PVP (MW ~40,000) showed also formation of 2-pyrrolidone only. MALDI-TOF-MS spectra of pure PVP and NRCWE-009 showed similar peak pattern and had the same repeating unit of m/z 111. Therefore it was concluded that the coating of NRCWE-009 is PVP [52]. The formed 2-pyrrolidone during PLE explained 12 wt.% of the TGA mass loss, but taking into consideration that a –CH_2_–CH- unit for each molecule of 2-pyrrolidone has disappeared during the thermal degradation the explained fraction adds up to 15 wt.%. However, it has previously been shown that thermal desorption combined with GC-MS can explain the entire TGA mass loss of NRCWE-009 if calibrated with PVP [52]. For NM-300K, which is a suspension in water, GC-MS showed traces of fatty acids C12-C18 (main component oleic acid) and polyethylene glycols (n = 2–3). The MALDI spectrum of NM-300K was complex, but apparently 5 series of polyethoxylates could be discerned. These observations were not contrary to the description of NM-300K [53] which was added 4 wt.% polyoxyethylene glycerol trioleate (Tagat^®^ TO) (see [56,64]) and 4 wt.% polyoxyethylene sorbitan monolaurate (Tween^®^ 20) (see [65]) as dispersion stabilizers. Thus MALDI-TOF-MS showed the polymeric nature of the associated organic compounds and GC-MS indicated the identity of the end groups. 

For the calcium carbonate (CaCO_3_) MNMs (NRCWE-012 to NRCWE-017) GC-MS results explained 12–68 wt.% of the TGA mass loss and were in all cases fatty acids and their methyl esters, except for NRCWE-016, which in addition contained 1,4-benzenedicarboxylic acid dimethyl ester and a high boiling mixture of a probably hydrated PAH. This mixture could have been added to make NRCWE-016 more compatible with rubber which it is intended for as an additive [66]. NRCWE-013, NRCWE-014, and NRCWE-015 were apparently also associated with a silica contamination indicated by the relatively large amounts of TMOS in the PLE extracts. Calcium carbonate particles are often surface treated with stearic acid [67] which may also change the toxicity [68].

The PLE extracts of neither the iron oxide (Fe_2_O_3_) MNMs (NRCWE-018 and NRCWE-019) nor the nickel-zinc-iron oxide (Ni/ZnFe_2_O_4_) MNMs (NRCWE-020 and NRCWE-022) did contain any possible coating compounds detected with any of the applied MS methods in spite that they showed TGA mass losses of 2–3 wt.%. A possible impurity content of iron hydroxides might explain mass loss at 200–480 °C by dehydration forming FeO(OH) [69]. But dehydration of trapped synthesis liquids and/or dehydroxylation is also a possibility as suggested above. However, it should also be noted that for all four MNM there was a content of TMOS in the extracts and might stem from a SiO_2_ coating or an impurity.

For the organoclay MNMs (Nanofil 5^®^, Nanofil 8^®^, Nanofil SE3000^®^) LC-MS results explained 5–14 wt.% of the TGA weight losses and consisted of mixtures of dilkyldimethyl-QAC. For Nanofil 9^®^ LC-MS explained 7 wt.% of the TGA mass loss and consisted of mixtures of alkyldimethylbenzyl-QAC. These results were in agreement with the information on the surface functionalization in the material safety data sheets. 

### 3.4. Summary Considerations

The purpose of this work was to demonstrate the applicability of using TGA (or water-loss and LOI by the laboratory furnace method) combined with extraction and MS analysis as an operational approach for quantification and identification of extractable organic surface coatings of a wide range of different MNM. 

The central idea of the proposed approach is that mass loss after thermal treatment (TGA or laboratory furnace method) can be used to identify possible presence of organic coatings and quantify the amount and that MS can be used to identify the chemical coating components. The approach is based on at least five assumptions: (1) the mass-loss adjusted for water loss during TGA (or LOI) is due to evaporation and combustion of organic compounds constituting the entire coating, (2) all components of the organic coating is extracted to a suitable solvent, (3) the composition of organic compounds found by one (1^st^) extraction is representative for the entire coating despite low recovery, (4) MS analysis detect and identify all components, (5) that the particle core is purely inorganic. 

Assumption 1: Quantification of organic surface coating on MNM by *TGA*. The TGA results indicated inhomogeneity of the analysed amounts of MNM both with respect to the amount of coating (corrected for water-loss) and the inorganic core itself. This means that the lesser the amount of test material the lesser representative it will be for the sample MNM. This is shown by relative larger standard error means for the TGA results of the coating amount as compared to laboratory furnace method (Table 2). However, on average the two methods are in compliance (Figure 2b). The largest differences are in the low amount range below 5% coating which includes most of the investigated MNM. It must be decided by specific assessment how exactly the amount of coating should be estimated dependent on the purpose of the analysis or study involving the MNM. If only small amounts of sample are available TGA is the only possibility. To make a proper quantification of the organic coating by TGA, it is of crucial importance to know the identity of the inorganic core to prevent erroneous results due to e.g. weight loss caused by decomposition as seen in the case of carbon and carbonates. It is also important to know presence and nature of impurities and additives that also may decompose or combust during thermal treatment. 

The underestimation of the water loss by TGA compared to the laboratory furnace method indicates that a lower TGA heating rate would make the two methods even more comparable which is taken into account in the NANoREG protocol for estimation of organic coating by TGA [44]. Alternatively, the TGA dwell-temperature could be raised to 110 °C to provide more activation energy for evaporation and/or the dwell-time at 100 °C or 110 °C could be extended beyond 10 min. This could improve driving out the water potentially trapped or adsorbed harder in nanoporous materials such as NM-101.

Assumption 2: All components of the organic coating are extracted. The lower the recovery of the extraction the more uncertain it is that all components of the coating have been extracted. Reasons for low recovery of PLE may include: Strong adsorption of coating molecules to the surface of the MNM, covalent bonding of coating molecules to the surface of the MNM (bonds not broken by the harsh PLE conditions), and ionic bonding of coating molecules to the surface of the MNM (organoclays).

Assumption 3: Composition of organic compounds found by one (1^st^) extraction is representative for the entire coating despite low recovery. Even when the chromatographic results indicate a low recovery by extraction it is assumed that the relative content or composition of compounds in the extract is constant and will be the same if the extraction was exhaustive. This statement is mainly based on the five consecutive extractions of QAC from the organoclays (Figure 3). If the surface coating is composed of several organic compounds with similar physico-chemical properties it is probably a fair assumption. Also if the coating molecules belong to the same family, e.g., siloxanes the assumption might be fair. However, if the coating molecules have very different physico-chemical properties and have different chemical functionalities the assumption may fail. When the assumption is used to give a quantitative estimate of identified coating compounds they should be normalized to the total amount of coating determined by TGA. Also, if no chromatographic peaks are observed and a polymeric compound is identified by MALDI-TOF-MS all coating is assumed to be this polymer and thus the total amount given by TGA. 

Assumption 4: Identification of the organic surface coating on MNM. Collection of all possible information on the MNM from manufacturer, suppliers, articles etc. is crucial as support for the MS identification process, which is not infallible. MNM with no obvious organic coating (<1 wt.%) estimated by TGA were omitted for further analysis except if the collected information indicates a possible coating or association with organic compounds. The rest of MNM with possible organic coating are extracted and analysed with GC-MS (volatile organic compounds) or LC-MS (non-volatile and polar organic compounds). If the extraction results together with TGA gives rise to suspicion of polymeric coating the MNM was analysed with MALDI-TOF-MS. 

Low recovery by extraction may result in identification of false coating molecules since it can be a background contamination including carry-over in the GC-MS system, contamination of the MNM, degradation products of the original coating if it is thermally labile (NRCWE-009) or fragments of covalently bonded coating molecules (NM-111).

Only for NM-103, NM-104, NM-111, NM-300K, and the organoclays more or less precise a priori information on the surface coating or stabilizer (NM-300K) was known. In the cases of NM-111 and NM-300K our MS analysis could confirm this information though they only explained a small fraction of the mass loss measured by TGA. In the case of NM-103 and NM-104 our MS analysis could not confirm the manufacturer’s information or published results.

Assumption 5: The particle core is purely inorganic. Both the TGA and the solvent extraction procedure results cannot be attributed to “coating“ components if assumption 5) is not fulfilled. This is the case for a large number of MNMs (especially organic pigments) that are reported to the French R-nano inventory, and which are on the EUON list of expected registrations under REACH. However, the elemental composition from bulk chemical analysis should reveal the inorganic or organic nature of the core.

If TGA indicates the existence of an organic coating though detection of any organic coating molecules utilizing extraction and MS methods is unsuccessful, it cannot be concluded that there is no organic coating. The amount of coating may, however, be low (<1 wt.%), the coating may not be extractable for example because it is covalently bound to surface or consist of polymeric compounds which cannot be ionized by MALDI-TOF-MS. A detector coupled to TGA would be important in order to be able to further conclude on the TGA measurements, especially to detect water loss for a better and more accurate estimation of water content and to distinguish between release of organic compounds, loosely bound water, dehydration of hydroxyl groups or crystal water. Surface spectroscopy techniques such as ATR-IR and XPS may supplement the TGA, extraction and MS methods as seen for NM-111. MS is probably the method, which can give the most unambiguous identification of an organic compound. XPS may give results on surface chemistry that may be difficult to interpret as the X-rays penetrate relatively deep (ca. 10 nm) into the material.

Finally, we would like to point out that we did not analyze a dedicated, synthesized series of e.g., different well-known coatings on the same core, but demonstrated the strategy on commercially available as well as representative nanomaterials from the test programme under the OECD Working Party on Manufactured Nanomaterials [70].

## 4. Conclusions

The presented approach for identification and quantification of extractable organic surface coating is relatively simple but cannot give a satisfactory answer for all types of MNM, i.e., TGA may indicate an organic coating while nothing can be detected by MS. However, the approach using TGA, extraction, separation, and subsequent MS was shown to be successful for 14 out of the 24 investigated MNMs. For 10 MNMs no coating compounds could be detected by MS, in spite of the fact that TGA showed mass losses of 2%–4%, presumably due to organic compounds. These MNMs need investigation using completely different analytical surface techniques such as XPS, ATR-IR, and EM.

## Figures and Tables

**Table 1:** Studies using mass spectrometry analysis of surface coating/functionalization of MNM.

MNM	Technique*	Comments	Ref.
Multi-functionalized Au	HPLC-MS-UV-CLND	Identified and quantified individual ligands	[30,31]
Mixed-monolayer coated Au	MALDI-TOF-MS	Semi-quantitative measure of ligand composition	[32]
Mixed thiolate coated Au	IMS-MS	Relative quantity of ligands	[33]
f-CNT	GC-MS	Chemical decoupling of ligands	[34]
f-CNT	TGA-FTIR-MS	Identification by FTIR-MS, quantification by TGA	[35]
Oleate coated magnetite (Fe^2+^Fe^3+^_2_O_4_)	TGA-MS		[36]
Functionalized FePt and Fe_3_O_4_	MALDI-TOF-MS and LC-MS	Chemical decoupling followed by LC-MS	[37]
Organic coated TiO_2_	ESI-MS and MALDI-TOF-MS	Extraction with PLE followed by MS	[38]
Organic coated ZnO, NiFe_2_O_4_, YYbErO_2_S, CNT	TOF-SIMS	Several other techniques were also applied	[39]
Organic coated CNT	TOF-SIMS	Several other techniques were also applied	[40]
Organic coated Ag, Au, Pd, PdAg, Fe, Ni, Cu	LV-AMS	Cannot be used in case of several unknown organic compounds	[41]

* HPLC = High Performance Liquid Chromatography; MS = Mass Spectrometry; UV = Ultra Violet spectrometry; CLND = chemiluminescent nitrogen detection; MALDI-TOF-MS = Matrix-Assisted Laser Desorption Ionization Time-Of-Flight MS; IMS-MS = Ion Mobility Spectrometry MS; GC-MS = Gas Chromatography MS; LC-MS = High Performance Liquid Chromatography MS; ESI-MS = ElectroSpray Ionization MS; TOF-SIMS = Time-Of-Flight Secondary Ion MS; LV-AMS = laser-vaporization aerosol MS.

**Table 2:** Available information on the MNMs, and measured water content and maximum amount of possible organic coating of the MNMs (mass loss before any decomposition of the inorganic core) by two different methods: TGA and the laboratory furnace method. The loss is %-w/w and the uncertainty is indicated by the standard error of mean (σ).

Substance	Code	Phase	Supplier [Reference]	Batch Number	Suppliers’ Information on Purity/Coating	Water Loss by TGA @25–110 °C (wt.%)	σ	Coating TGA >110 °C (wt.%)	σ	Water Loss Furnace @110 °C (wt.%)	σ	Coating Furnace >110 °C (wt.%)	σ
TiO_2_	M111	Rutile	Kemira [45]	-	-	3.8	0.19	4.08	0.2	4.86	0.01	5.72	0.04
	NM-101	Anatase	JRC [46]	-	-	8.21	0.69	4.41	0.35	-	-	-	-
	NM-103	Rutile	==||==	-	6wt.%Al_2_O_3_; 2wt.% Dimethicone	1.62	0.16	2.54	0.24	-	-	-	-
	NM-104	Rutile	[47] Suppl. Mat.	-	6wt.%Al_2_O_3_; 1wt.% glycerin	1.49	0.1	3.17	0.07	-	-	-	-
SiO_2_	NM-204	^£^SAS	JRC [48]	-	-	4.44	0.38	3.14	0.34				
	NRCWE-008	^£^SAS	NanoAmor [49]	4850MR	-	3.13	0.98	4.49	0.49	5.86	0.02	4.19	0.1
Graphite	NRCWE-005	^$^Graphite	SS Nano [50]	0520BX	-	25.38	1.47	2.68	0.28	3.13	0.48	-	-
ZnO	NM-111	Zincite	JRC [51]	-	Triethoxyoctyl silane	0	-	2.1	0.31	-	-	-	-
Ag	NRCWE-009	Ag(m)	NANOGAP [52]	NP Ag-2103	85wt.% Ag*	1.6	0.2	14.66	0.55	1.99	0.17	13.5	0.26
	NM-300K	Ag(m)	JRC [53]	-	Dispersion (see text)	67.62	1.32	15.95	0.43	-	-	-	-
CaCO_3_	NRCWE-012	^€^Calcite	SS Nano [50]	1952RH	Yes; < 0.5 wt.% H_2_O	0.43	0.11	5.33	0.11	0.46	0.03	4.7	0.03
	NRCWE-013	^€^Calcite	==||==	1953RH	Yes; < 0.5 wt.% H_2_O	0.54	0.22	5.72	0.08	0.5	0.12	4.8	0.12
	NRCWE-014	^€^Calcite	==||==	1954RH	Yes; < 0.5 wt.% H_2_O	0.24	0.08	5.09	0.3	0.43	0.01	4.66	0.02
	NRCWE-015	^€^Calcite	==||==	1955RH	Yes; < 1.2 wt.% H_2_O	0.64	0.15	5.5	0.12	0.87	0.05	5.96	0.05
	NRCWE-016	^€^Calcite	==||==	1956RH	Yes; < 0.5 wt.% H_2_O	0.15	0.03	4.75	0.24	0.53	0.02	5.28	0.02
	NRCWE-017	^€^Calcite	==||==	1957RH	< 0.5 wt.% H_2_O	0.34	0.02	5.52	0.18	0.61	0.03)	5.33	0.08
Fe_2_O_3_	NRCWE-018	α-Fe_2_O_3_ particle	NanoAmor [49]	2520ZH	99 wt.% purity	0.69	0.15	1.97	0.11	1.02	0.07	3.1	0.06
	NRCWE-019	α-Fe_2_O_3_ rod	==||==	8004NJ	None	0.76	0.26	2.19	0.37	2.28	0.06	3.12	0.06
Ni/ZnFe_2_O_4_	NRCWE-020	Ni_0.5_Zn_0.5_Fe_2_O_4_	NanoAmor [49]	4115FY	98.5 wt.% purity	2.63	0.17	3.03	0.15	2.74	0.02	3.28	0.03
	NRCWE-022	NiFe_2_O_4_	==||==	4110FY	98 wt.% purity	2.81	0.17	3.04	0.33	2.61	0.03	3.21	0.04
Organoclay	Nanofil^®^5	Organoclay	Südchemie	-	ca. 35 wt.% QAC	2.24	0.18	35.12	0.24	1.22	0.03	35.47	0.04
	Nanofil^®^8	Organoclay	Südchemie	-	ca. 45 wt.% QAC	0.99	0.51	44.09	0.35	1.46	0.03	43.74	0.03
	Nanofil^®^9	Organoclay	Südchemie	-	ca. 35 wt.% QAC	1.47	0.3	34.89	0.4	1.1	0.01	37.04	0.01
	Nanofil^®^SE3000	Organoclay	Südchemie	-	unknown QAC	1.13	0.2	53.75	0.41	0.69	0.004	55.04	0.11

^£^ SAS: Synthetic Amorphous Silica; ^$^ 93 wt.% graphite; ^€^ > 94.5 wt.% purity; * Special request.

**Table 3:** Identified and unidentified major organic compounds and groups associated with the MNMs in Table 2 given as semi-quantitative % (w/w) of MNM mass. A number of un-identified GC-MS peaks are not listed but included in “Sum of un-identified compounds”. Un-identified compounds are marked with “?”. Tentatively identified compounds (marked with *) have not been verified by authentic standards. Results on single compound level are shown in Table S2 and illustrative chromatograms in Figure S2

	Code	Chromatography	Mass Spectrometry	Trimethoxymethylsilane	Tetramethoxy silane, TMOS (Artifact not Added to the Sum of Compounds)	Hexamethyl cyclotrisiloxane	Silane?	Silane?	2-pyrrolidone^B^	Trimethoxyoctylsilane	1,4-Benzenedicarboxylic acid dimethylester*	Aromatic Compound? Base Peak m/z = 269	ΣFatty acid methyl esters (C8 – C18)	ΣFatty acids (C8 – C18)	Large Peak Cluster (probably Reduced (hydrated) PAH Mixture)	Sum of GC-MS un-identified Compounds (%)	Sum of GC-MS Identified and Tentatively Identified Compounds (%)	ΣDilkyldimethylammonium Compounds (C7 - C27, Mainly C18, C16, C14) by LC-MS	ΣAlkyldimethylbenzylammonium Compounds (C14 - C21, Mainly C18, C16, C14) by LC-MS	MALDI-TOF-MS results	Fraction of TGA Coating Explained by Extraction and GC- or LC-MS (%)
	GC-MS retention (min)			3.2	4.9	6.4	7.5	14.6	15.3	24.4	27.3	31.6	17–36	17–36	32–42						
TiO_2_	UV-Titan M111	GC	MS^$^													0	0				0
	NM-101	GC	MS, MALDI													0	0			^A^0	0
	NM-103	GC, LC	MS, QTOF, MALDI	9 × 10^–4^	4 × 10^–2^	9 × 10^–4^	3 × 10^–2^	1 × 10^–2^								4 × 10^–2^	2 × 10^–3^			^B^3 × 10^–2^	3
	NM-104	GC, LC	MS, QTOF, MALDI		6 × 10^–3^											0	0			0	0
SiO	NM-204	GC	MS, MALDI		2 × 10^–1^											0	0			0	0
	NRCWE-008	GC	MS, MALDI		^E^13											0	0			0	0
Graphite	NRCWE-005	GC	MS, MALDI		2 × 10^–2^					4 × 10^–4^		6 × 10^–6^	2 × 10^–2^	1 × 10^–1^		6 × 10^–4^	2 × 10^–1^			0	6
ZnO	MN-111	GC	MS							2 × 10^–1^						0	2 × 10^–1^				10
Ag	NRCWE-009	GC	MS, MALDI						2							7 × 10^–2^	2			C	12
	NM-300K (liquid)	GC	MS, MALDI																	D	
CaCO3	NRCWE-012	GC	MS									2 × 10^–4^	5 × 10^–1^	2		2 × 10^–4^	3				52
	NRCWE-013	GC	MS		1 × 10^–1^								3^–1^	4^–1^		0	7^–1^				12
	NRCWE-014	GC	MS		5 × 10^–2^								2 × 10^–1^	1		0	1				27
	NRCWE-015	GC	MS		1 × 10^–1^								3 × 10^–2^	4		0	4				68
	NRCWE-016	GC	MS								8 × 10^–3^		3 × 10^–2^	1	6 × 10^1^	6 × 10^–1^	1				36
	NRCWE-017	GC	MS									1 × 10^–4^	5 × 10^–1^	3		1 × 10^–4^	3				60
Fe_2_O_4_	NRCWE-018	GC, LC	MS, QTOF, MALDI		8 × 10^–2^											0	0			0	0
	NRCWE-019	GC, LC	MS, QTOF, MALDI		4 × 10^–2^											0	0			0	0
Ni/ZnFe_2_O_4_	NRCWE-020	GC, LC	MS, QTOF, MALDI		1 × 10^–2^			00A0								0	0			0	0
	NRCWE-022	GC, LC	MS, QTOF, MALDI		2 × 10^–2^											0	0			0	0
Organoclay	Nanofil 5^®^	LC	QTOF^£^															3.3			9
	Nanofil 8^®^	LC	QTOF															6.1			14
	Nanofil 9^®^	LC	QTOF																2.3		7
	Nanofil SE3000^®^	LC	QTOF															2.6			5

$ MS = electron ionization MS. £ QTOF = electrospray ionization quadrupole time of flight MS. * = tentatively identified. A: No polymeric compounds detected by MALDI-TOF-MS. B: LC-MS MS showed series of polyethoxylates confirmed by MALDI-TOF-MS, C: Polyvinylpyrrolidone which forms 2-pyrrolidone as thermal degradation product which in turn can was used to quantify the amount of coating [57]. D: Different polyethoxylates (see text). E: The maximum measured amount of tetramethoxysilane in the extracts of NRCWE-008.

## Supplementary Materials

The following are available online at https://www.mdpi.com/1996-1944/12/22/3657/s1, Figure S1: TGA curve of NM-300K., Figure S2: Illustrative chromatograms of extracts of the different types of MNM, Figure S3: Water loss of MNM estimated by TGA from 25–110 °C at 10 °C/min versus the laboratory furnace method. Table S1: Available information on the studied MNM, Table S2: Identified and un-identified organic compounds associated with MNMs.
