# Supplementary material for: Thermogravimetry and Mass Spectrometry of Extractable Organics from Manufactured Nanomaterials for Identification of Potential Coating Components
Source: Materials (Basel). 2019 Nov 6;12(22):3657. doi: 10.3390/ma12223657 (PMC6888238; doi:10.3390/ma12223657)
Supplement: Supplementary file 1 [file materials-12-03657-s001.pdf]

Supplementary material:

Identification and quantification of extractable organic surface coating on manufactured nanomaterials by thermogravimetry and mass spectrometry

Per Axel Clausen, Vivi Kofoed-Sørensen, Asger W. Nørgaard, Nicklas Mønster Sahlgren, Keld Alstrup Jensen

National Research Centre for the Working Environment, Denmark.

Table S1. Available information on the studied MNM

	Code	Phase	Application	Manufacturer/ supplier/Reference	Primary particle size	BET, m^2/g	Manufacturer's information on impurity / coating
TiO_2	UV-Titan M111	TiO_2	UV-Titan M111	Kemira [1]	14 nm		4.5% by TGA
	NM-101	TiO_2 (Anatase)	Semiconductor catalyst for use in photocatalytic processes	JRC [2]	5 nm	234	8% by TGA
	NM-103	TiO_2 (Rutile)	Cosmetics (sun care, colour), pharma, food	==||==	20-100 nm	52	Al_2O_3 6% - Dimethicone 2%
	NM-104	TiO_2 (Rutile)	Cosmetics (sun care, colour), pharma, food	==||==	8-200 nm	57	Al_2O_3 6% - Dimethicone 2%
Si	NM-204	SiO_2	Food	JRC [3]	10-15 nm	132	0.5% by TGA
	NRCWE-008	SiO_2		NanoAmor [4]	15 nm	65	?
	NRCWE-005	Graphite		SS Nano [5]	3-4 nm	225	5.5% by TGA
ZnO	NM-111	ZnO	Representative MNM	JRC [6]			Triethoxyoctyl silane coating
Ag	NRCWE-009	Ag		NANOGAB [7]	43±15 nm	0.03	17% by TGA
	NM-300K	Ag	Reference nanomaterial	JRC [8]	< 20 nm		Dispersion stabilizers (see text).
$CaCO_3$	NRCWE-012	$CaCO_3$	Water-based latex paint	SS Nano [5]	15-40 nm	22	4% by TGA
	NRCWE-013	$CaCO_3$	PVC plastics	==||==	15-40 nm	26	4% by TGA
	NRCWE-014	$CaCO_3$	PE/PP plastics	==||==	15-40 nm	26	4% by TGA
	NRCWE-015	$CaCO_3$	Printing inks	==||==	15-40 nm	32	4.5% by TGA
	NRCWE-016	$CaCO_3$	Rubber	==||==	15-40 nm	28	5% by TGA
	NRCWE-017	$CaCO_3$	Adhesives	==||==	15-40 nm	21	4% by TGA
Fe_2O_3	NRCWE-018	Fe_2O_3	Alfa-Fe_2O3 particle	NanoAmor [4]	20-60 nm	28	2.5% by TGA
	NRCWE-019	Fe_2O_3	Alfa-Fe_2O3 fiber	==||==	40-150 nm x 250-600 nm	27	2% by TGA
Ni/ZnFe	NRCWE-020	$Ni_{0.5}Zn_{0.5}Fe_2O_4$	Nickel-Zinc Iron Oxide	NanoAmor [4]	10-30 nm	104	?
	NRCWE-022	$NiFe_2O_4$	Nickel Iron Oxide	==||==	20-30 nm	87	?
Organoclay	Nanofil 5®	Organoclay		Südchemie	1 nm x 100-500 (8 µm)	8.4	ca. 35% QAC
	Nanofil 8®	Organoclay		Südchemie	1 nm x 100-500 (8 µm)	5.6	ca. 45% QAC
	Nanofil 9®	Organoclay		Südchemie	1 nm x 100-500 (8 µm)	24.1	ca. 35% QAC
	Nanofil SE3000®	Organoclay		Südchemie	1 nm x 100-500 (8 µm)	2.7	QAC

Table S2. Identified and un-identified organic compounds associated with MNMs. Semi-quantitative % of MNM mass. A number of un-identified GC-MS peaks are not listed but included in "Sum of un-identified compounds". Tentatively identified compounds have not been verified by authentic standards and are marked with *.

	Code	Chromatography	Mass Spectrometry	Trimethoxymethylsilane	Tetramethoxy silane (In no cases added to the sum of compounds)	Hexamethyl cyclotrisiloxane	Silane? (149,179,119,59,82,75,133,89)	Silane? (253,223,207)	2-pyrrolidinone (2-pyrrolidone)[B]	Octanoic acid methyl ester	Octanoic acid	Decanoic acid methyl ester	Trimethoxyoctyl silane (121,91,61)	1,4-Benzenedicarboxylic acid dimethylester *	Dodecanoic acid methyl ester	Dodecanoic acid	Tetradecanoic acid methyl ester	Tetradecanoic acid	Aromatic compound? Base peak m/z = 269	Pentadecanoic acid	Hexadecanoic acid methyl ester	Hexadecanoic acid	Octadecenoic acid methyl ester	Octadecanoic acid methyl ester	Octadecanoic acid	ΣFatty acid methyl esters (C8 – C18)	ΣFatty acids (C8 – C18)	Large Peak cluster (probably reduced (hydrated) PAH mixture)	Sum of GC-MS un-identified compounds (%)	Sum of GC-MS identified and tentatively identified compounds (%)	Dilkyldimethylammonium compounds (C7 - C27 ; mainly C18, C16, C14)	Alkyldimethylbenzylammonium compounds (C14 - C21 ; mainly C18, C16, C14)	LC-MS and MALDI results	Fraction of TGA coating explained by extraction and chromatography/MS (%)
	GC-MS retention time (min)			3.2	4.9	6.4	7.5	14.6	15.3	16.7	17.0	23.4	24.4	27.3	27.6	28.3	30.8	31.3	31.6	32.6	33.4	33.9	35.5	35.8	36.3	16.7-35.8	17.0-36.3	32-42						
TiO_2	UV-Titan M111	GC	MS																										0	0				0
	NM-101	GC	MS, MALDI																										0	0			0[B]	0
	NM-103	GC, LC	MS, QTOF, MALDI	9E-4	4E-2	9E-4	3E-2	1E-2																					4E-2	2E-3			3E-2[B]	3
	NM-104	GC, LC	MS, QTOF, MALDI		6E-3																								0	0			0	0
SiO_2	NM-204	GC	MS, MALDI		2E-1																								0	0			0	0
	NRCWE-008	GC	MS, MALDI		13[E]																								0	0			0	0
Graphite	NRCWE-005	GC	MS, MALDI		2E-2					7E-4	7E-4	3E-4	4E-4		5E-3	8E-2	2E-3	3E-2	3E-6		6E-3	4E-2	6E-4	9E-3		2E-2	1E-1		6E-4	2E-1			0	6
ZnO	MN-111	GC	MS										2E-1																0	2E-1				10
Ag	NRCWE-009	GC	MS, MALDI						2																				7E-2	2			C	12
	NM-300K (liquid)	GC	MS, MALDI																										0	0			D	
$CaCO_3$	NRCWE-012	GC	MS												1E-3		2E-3	4E-2	2E-4	7E-2	3E-1	2		2E-1	6E-1	5E-1	2		2E-4	3				52
	NRCWE-013	GC	MS		1E-1										9E-5		2E-3				1E-1	3E-1		1E-1	2E-1	3E-1	4E-1		0	7E-1				12
	NRCWE-014	GC	MS		5E-2												1E-3				7E-2	9E-1		1E-1	3E-1	2E-1	1		0	1				27
	NRCWE-015	GC	MS		1E-1											2E-1	2E-4	1E-1			2E-2	2		2E-2	2	3E-2	4		0	4				68
	NRCWE-016	GC	MS											8E-3							1E-2	4E-1		2E-2	7E-1	3E-2	1	6E-1	6E-1	1				36
	NRCWE-017	GC	MS												7E-4		2E-3	9E-2	1E-4		3E-1	2		2E-1	7E-1	5E-1	3		1E-4	3				60
Fe_2O_3	NRCWE-018	GC, LC	MS, QTOF, MALDI		8E-2																								0	0			0	0
	NRCWE-019	GC, LC	MS, QTOF		4E-2																								0	0			0	0
$Ni/ZnFe_2O_4$	NRCWE-020	GC, LC	MS, QTOF, MALDI		1E-2																								0	0			0	0
	NRCWE-022	GC, LC	MS, QTOF, MALDI		2E-2																								0	0			0	0
Organoclay	Nanofil 5®	LC	QTOF																												3.3		D	9
	Nanofil 8®	LC	QTOF																												6.1		D	14
	Nanofil 9®	LC	QTOF																													2.3	D	7
	Nanofil SE3000®	LC	QTOF																												2.6		D	5

[$] MS = electron ionization MS. [£] QTOF = electrospray ionization quadrupole time of flight MS. * = tentatively identified A: No polymeric compounds detected by MALDI-TOF-MS. B: LC-MS MS showed series of polyethoxylates confirmed by MALDI-TOF-MS, C: Polyvinylpyrrolidone which forms 2-pyrrolidinone as thermal degradation product which in turn can was used to quantify the amount of coating [7]. D: Different polyethoxylates. E: The maximum measured amount of tetramethoxysilane in the extracts of NRCWE-008.

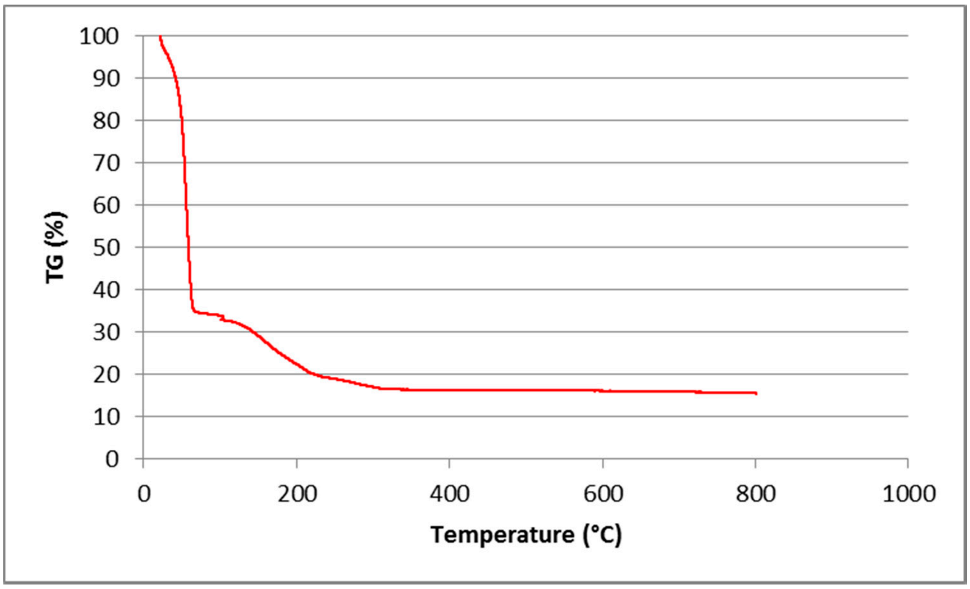

Figure S1. TGA curve of NM-300K.

Figure S2. Illustrative chromatograms of extracts of the different types of MNM

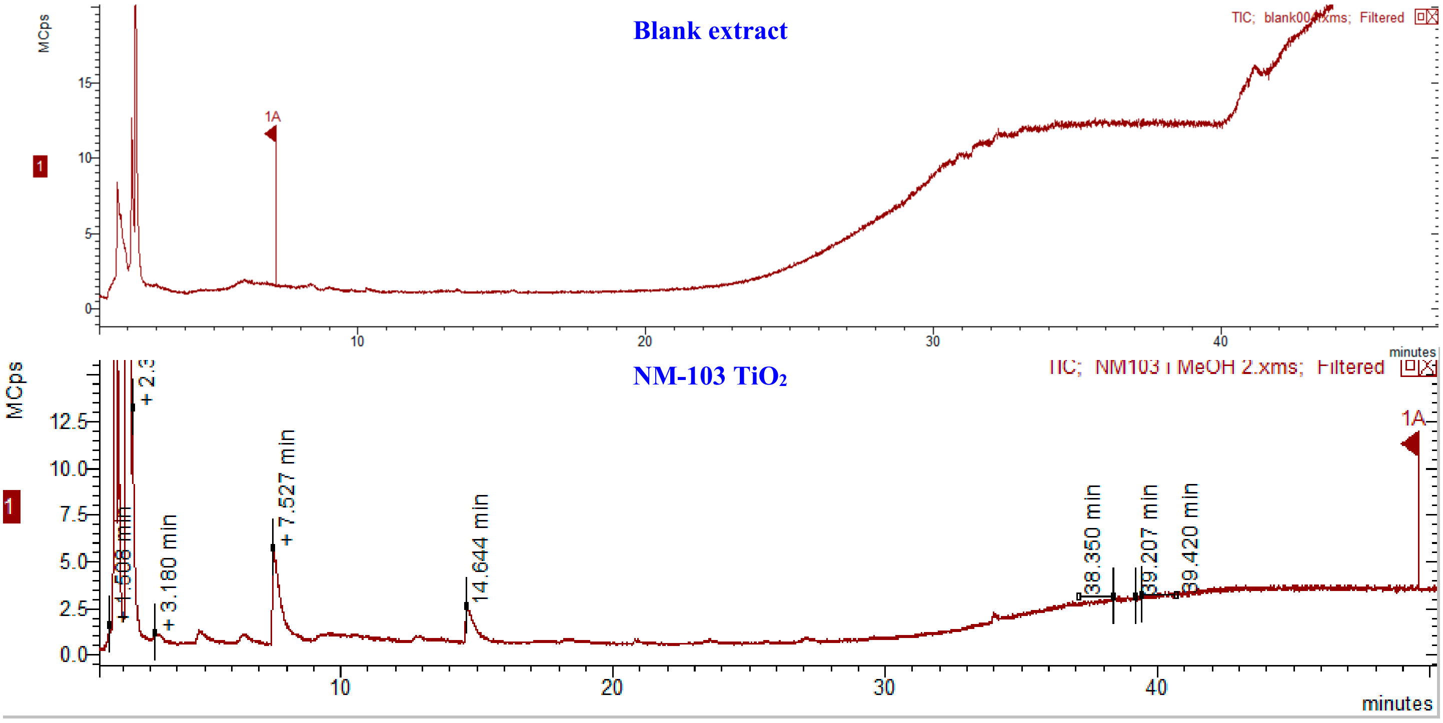

NRCWE-008 SiOx

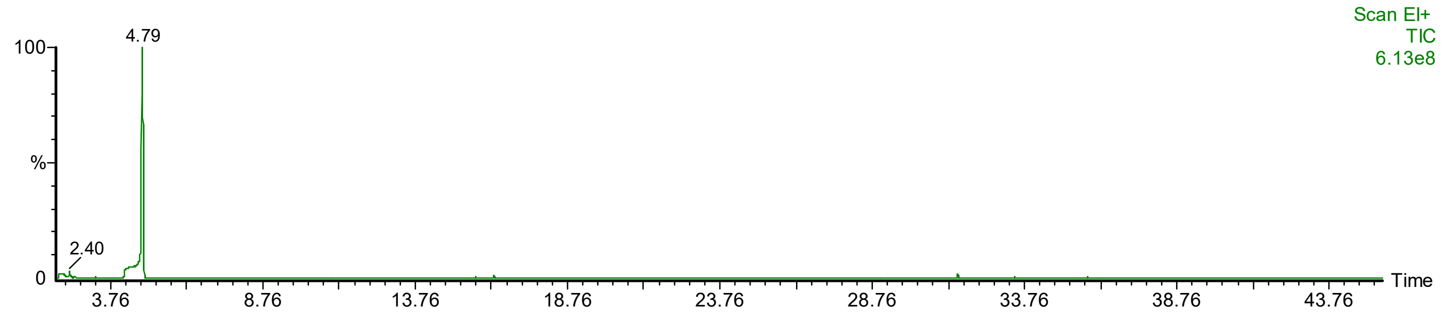

Prove 5 NRCWE-005 Graphite NP

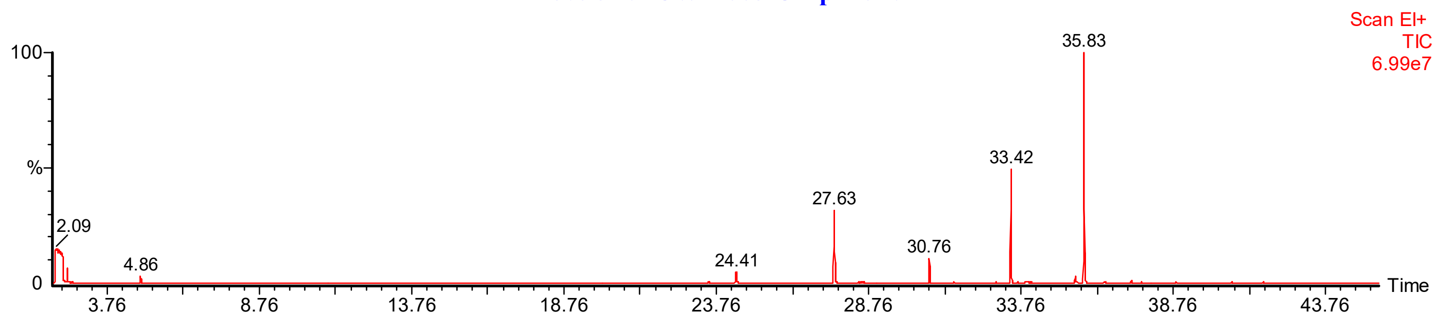

NM-111 ZnO

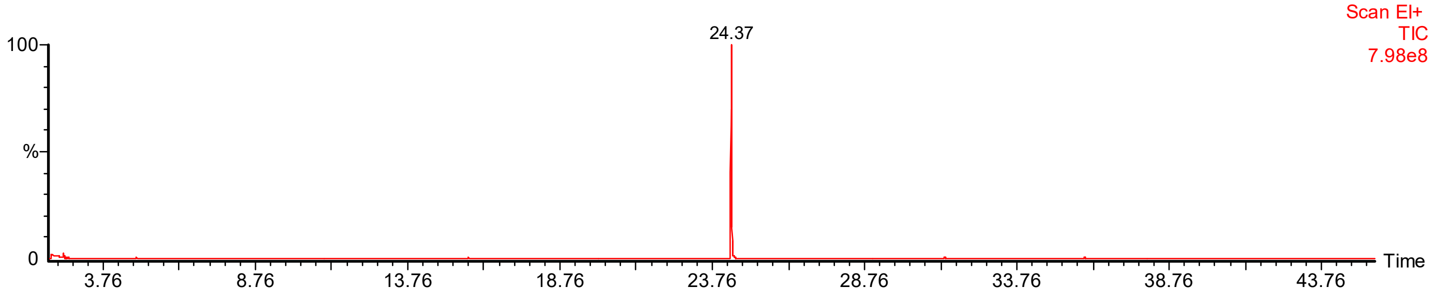

NRCWE-009 Nanogab

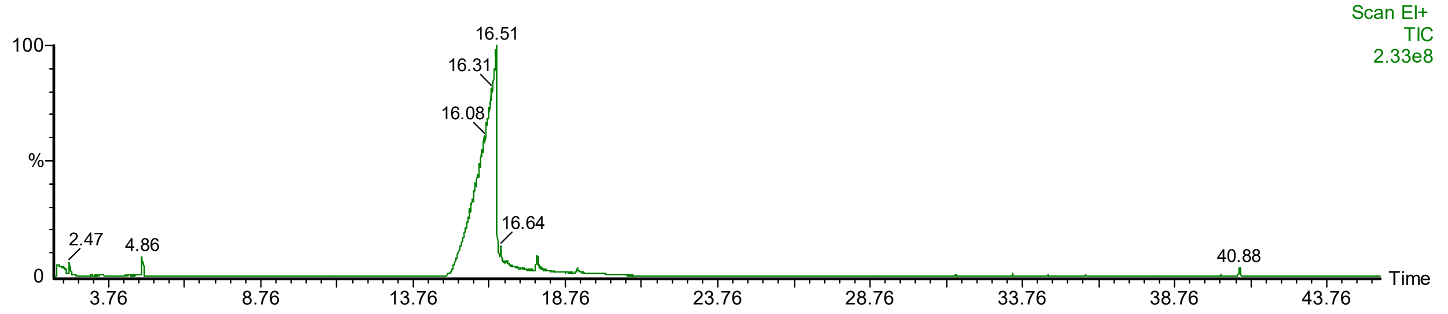

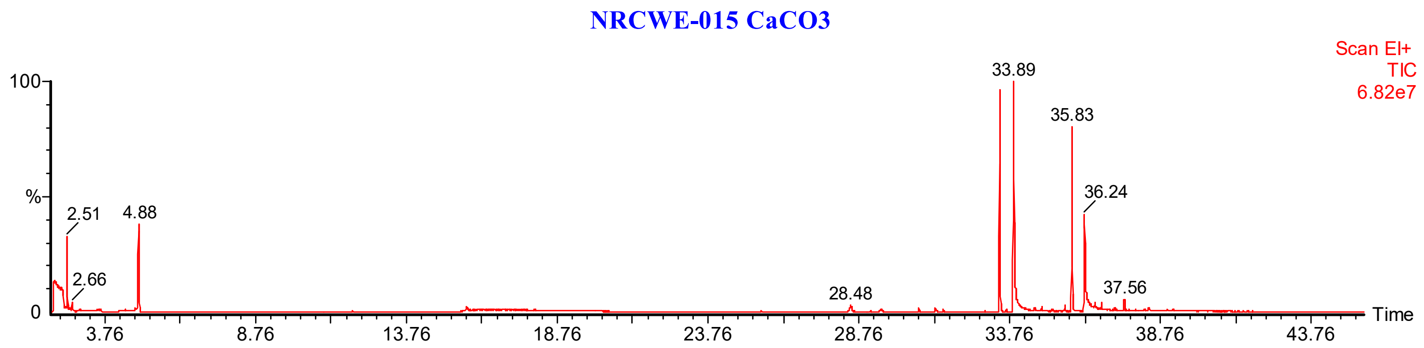

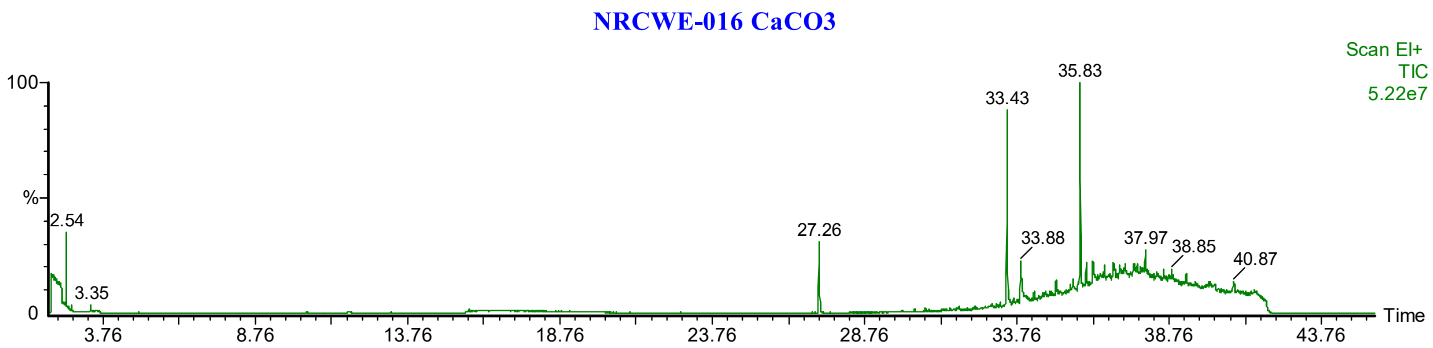

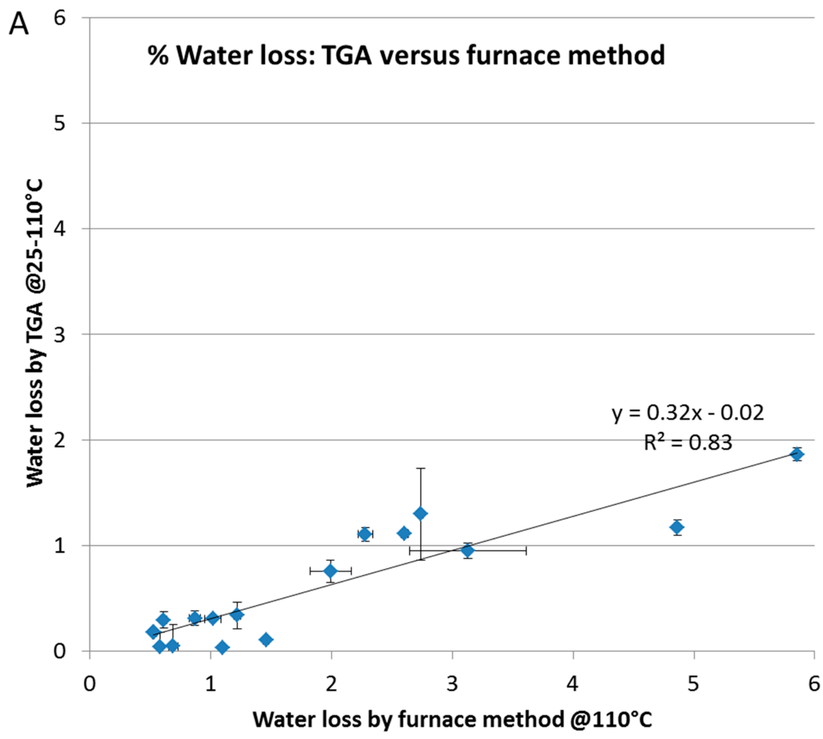

Figure S3. Water loss of MNM estimated by TGA from 25-110 °C at 10°C/min versus the laboratory furnace method. The error bars represent the standard error of mean (SEM).

References

1. SpecialChem Homepage UV-Titan M111. https://cosmetics.specialchem.com/product/i-sachtleben-uv-titan-m111
2. Rasmussen, K.; Mast, J.; De Temmerman, P.-J.; Verleysen, E.; Waegeneers, N.; Van Steen, F.; Pizzolon, J. C.; De Temmerman, L.; Van Doren, E.; Jensen, K. A.; Birkedal, R. K.; Levin, M.; Nielsen, S. H.; Koponen, I. K.; Clausen, P. A.; Kofoed-Sørensen, V.; Kembouche, Y.; Thieriet, N.; Rousset, D.; Spalla, O.; Guiot, C.; Rousset, D.; Witschger, O.; Bau, S.; Bianchi, B.; Motzkus, C.; Shivachev, B.; Dimowa, L.; Nikolova, R.; Nihtianova, D.; Tarassov, M.; Petrov, O.; Bakardjieva, S.; Gilliland, D.; Pianella, F.; Ceccone, G.; Spampinato, V.; Cotogno, G.; Gibson, N.; Gaillard, C.; Mech, A. *Titanium Dioxide, NM-100, NM-101, NM-102, NM-103, NM-104, NM-105: Characterisation and Physico-Chemical Properties*; EUR 26637 EN; Luxembourg, 2014, 2014; pp 1-208. DOI, http://dx.doi.org/10.2788/79554
3. Rasmussen, K.; Mech, A.; Mast, J.; De Temmerman, P.-J.; Van Doren, E.; Waegeneers, N.; Van Steen, F.; Pizzolon, J. C.; De Temmerman, L.; Jensen, K. A.; Birkedal, R. K.; Levin, M.; Nielsen, S. H.; Koponen, I. K.; Clausen, P. A.; Kembouche, Y.; Thieriet, N.; Rousset, D.; Bau, S.; Bianchi, B.; Witschger, O.; Spalla, O.; Giout, C.; Shivachev, B.; Gilliland, D.; Pianella, F.; Gibson, P. N.; Rauscher, H.; Ceccone, G.; Cotogno, G.; Stamm, H. *Synthetic Amorphous Silicon Dioxide (NM-200, NM-201, NM-202, NM-203, NM-204). Characterisation and Physico-Chemical Properties*; EUR 26046 EN; Luxembourg, 2013, 2013; pp 1-208. DOI, DOI 10.2788/57989
4. Nanostructured_&_Amorphous_Materials_(NanoAmor) Homepage. https://www.nanoamor.com/home
5. Skyspring_Nanomaterials Catalog - Nanomaterials from Skyspring. https://ssnano.com/i/u/10035073/h/CAT/Catalog_SSNano-2015.pdf
6. Singh, C.; Friederics, S.; Levin, M.; Birkedal, R.; Jensen, K. A.; Pojana, G.; Wohlleben, W.; Schulz, S.; Wiench, K.; Turney, T.; Koulaeva, O.; Marshall, D.; Hund-Rinke, K.; Kördel, W.; Van Doren, E.; De Temmerman, P.-J.; Francisco, M. A. D.; Mast, J.; Gibson, N.; Koeber, R.; Linsinger, T.; Klein, C. L., NM-Series of representative manufactured nanomaterials: Zinc Oxide NM-110, NM-111, NM-112, NM-113 - Characterization and test item preparation. *EUR 25066 EN - 2011* **2011**. DOI,
7. Nymark, P.; Catalán, J.; Suhonen, S.; Järventaus, H.; Birkedal, R.; Clausen, P. A.; Jensen, K. A.; Vippola, M.; Savolainen, K.; Norppa, H., Genotoxicity of polyvinylpyrrolidone-coated silver nanoparticles in BEAS 2B cells. *Toxicology* **2013,** *313*, 38-48. DOI, http://dx.doi.org/10.1016/j.tox.2012.09.014
8. Klein, C. L.; Comero, S.; Stahlmecke, B.; Romazanov, J.; Kuhlbusch, T.; Van Doren, E.; De Temmerman, P. J.; Mast, J.; Wick, P.; Krug, H., NM-Series of representative manufactured nanomaterials: NM-300 Silver characterisation, stability, homogeneity. *EUR 24693 EN-2011* **2011**. DOI,
